# Diagnostic Yield of Genetic Testing in Sudden Cardiac Death with Autopsy Findings of Uncertain Significance

**DOI:** 10.3390/jcm10091806

**Published:** 2021-04-21

**Authors:** Mercedes Iglesias, Tomas Ripoll-Vera, Consuelo Perez-Luengo, Ana Belen García, Susana Moyano, Juan Carlos Canos, Juan Carlos Borondo, Jorge Alvarez, Damian Heine-Suñer, Bernardino Barcelo

**Affiliations:** 1 Cardiology Department, Son Llatzer University Hospital, IdISBa, CIBEROBN, 07198 Palma de Mallorca, Spain; mechiigl@hotmail.com (M.I.); jalvarezr@hsll.es (J.A.); 2 Balearic Institute of Legal Medicine, 07003 Palma de Mallorca, Spain; consuelo.perezluengo@justicia.es (C.P.-L.); anabelen.garcia@justicia.es (A.B.G.); 3 Histopathology Department, National Institute of Toxicology and Forensic Science, 08002 Barcelona, Spain; susana.moyano@justicia.es (S.M.); juancarlos.canos@justicia.es (J.C.C.); juancarlos.borondo@justicia.es (J.C.B.); 4 Department of Molecular Diagnosis and Clinical Genetics, Son Espases University Hospital, IdISBa, 07120 Palma de Mallorca, Spain; damian.heine@ssib.es; 5 Department of Clinical Analysis and Toxicology, Son Espases University Hospital, IdISBa, 07120 Palma de Mallorca, Spain; bernardino.barcelo@ssib.es

**Keywords:** sudden death, autopsy, genetic testing

## Abstract

Background: Sudden death (SD) in the young usually has an underlying genetic cause. In many cases, autopsy reveals unspecific and inconclusive results, like idiopathic left ventricular hypertrophy (LVH), nonsignificant coronary atherosclerosis (CA), and primary myocardial fibrosis (PMF). Their pathogenicity and their relation to SD cause is unknown. This study aims to evaluate the diagnostic yield of genetic testing in these cases. Methods: SD cases, between 1 and 50 years old, with findings of uncertain significance (idiopathic LVH, nonsignificant CA and PMF) on autopsy were evaluated prospectively, including information about medical and family history and circumstances of death. Genetic testing was performed. Results: In a series of 195 SD cases, we selected 31 cases presenting idiopathic LVH (*n* = 16, 51.61%), nonsignificant CA (*n* = 17, 54.84%), and/or PMF (*n* = 24, 77.42%) in the autopsy. Mean age was 41 ± 7.2 years. Diagnostic yield of genetic test was 67.74%, considering variants of unknown significance (VUS), pathogenic variants (PV) and likely pathogenic variants (LPV); 6.45% including only PV and LPV. Structural genes represented 41,93% (*n* = 13) of cases, while 38,7% (*n* = 12) were related to channelopathies. Conclusion: Molecular autopsy in SD cases between 1 and 50 years old, with findings of uncertain significance, has a low diagnostic yield, being VUS the most frequent variant observed.

## 1. Introduction

Sudden death (SD) is a serious event that has a great socioeconomic impact on families and the community, and its incidence increases with age. SD is defined as an unexpected death in an apparently healthy individual or carrier of a known disease, within the first hour of symptom onset, or when the person has been last seen alive and healthy within the previous 24 h [1].

SD has a prevalence of 20% [2] and an annual estimated incidence of 1.3–8.5/100,000 in Australia and New Zealand 3.5/100,000 in the United States, and 7/100,000 in Europe [3,4,5].

The most frequent cause of sudden death in adults above 35 years old is coronary disease [6,7], while it is genetic disorders in the younger population, including myocardium disease and channelopathies [8].

The relevance of achieving a correct diagnosis of the cause of SD is related to the opportunity to undertake family screening and provide advice for future death prevention when a genetic cause is identified [8].

Multidisciplinary teams have been developed for the correct evaluation of the SD event, including the forensic pathologist, cardiologist, pathologist, and geneticist, among others [9]. In cases in which a definite diagnosis is not met by traditional autopsy methods (histological and toxicological examination), genetic screening is added, what is known as molecular autopsy [10].

It is estimated that 31% of SD cases have no clear diagnosis [10,11]. Among these, there is a subgroup with autopsy findings of uncertain significance [12]. These include idiopathic left ventricular hypertrophy (LVH) in the absence of myocyte disarray or secondary causes [11,13,14]; coronary atherosclerosis (CA) without significant narrowing of the arterial lumen, defined as an obstruction inferior to 75% of the lumen of the vessel, along with no evidence of acute or chronic ischemia; primary myocardial fibrosis (PMF) without signs of structural or ischemic cardiopathy [11,14].

It is in these cases in which we aim to evaluate the usefulness of molecular autopsy to reach a precise diagnosis, considering that these findings of uncertain significance could be an innocent and coincidental finding, or part of an abnormal variant: physiological LVH in genetically predisposed individuals, or part of the HCM spectrum in the case of idiopathic LVH; or an ischemic trigger to unmask lethal arrhythmia of an underlying genetic cause in relation to nonsignificant CA and PMF [11,14].

## 2. Materials and Methods

### 2.1. Study Setting

SD cases registered in the MUSIB program (MUerte Súbita Islas Baleares) were included in a prospective study from February 2015 to January 2020. The program consists of a collaborative and multidisciplinary team between the Son Llatzer University Hospital and the Balearic Institute of Legal Medicine (Mallorca, Spain) for the complete analysis of SD cases in the young, including cardiologists, forensic pathologists, pathologists, geneticists, biologists, and chemists, to study nontraumatic SD in individuals from 1 to 50 years old.

All cases of SD included in the study had undergone a full cardiac autopsy by locally recognised expert pathologists, including histological and toxicological analysis. Then, they were classified into subgroups: autopsy findings of uncertain significance; autopsy findings diagnostic of cardiomyopathy; cases with no findings on autopsy. Mutation analysis was performed using “next generation sequencing” (NGS) [15], most of them with exome sequencing, selecting for the analysis those genes related to SD (“clinical exome”). The number of genes studied ranged from 194 to 380. Cases in which an extra-cardiac cause of death was identified were excluded from genetic testing. Samples were analyzed by Health in code^®^ (La Coruña, Spain), Imegen^®^ (Valencia, Spain) and Centogene^®^ (Rostock, Germany) until 2017 and from then onwards, by the genetic lab at Son Espases Hospital (Mallorca, Spain). Variants were filtered by a pre-established protocol, mainly based on the probable functional impact on the protein and the allele frequency. Predictive bioinformatics “in silico” tools were used. We applied the “American College of Medical Genetics and Genomics and the Association for Molecular Pathology” (ACMG/AMP) [16] consensus guidelines to classify variants as pathogenic (PV), likely pathogenic (LPV) or as variants of unknown significance (VUS), excluding those considered nonpathogenic, probably nonpathogenic and VUS with a frequency rate ≥0.02% in the GnomAD, ClinVar and ExAc databases, or those for which cosegregation could not be proven in studied cases. The program also establishes the evaluation of all first-degree relatives of the decedents. However, results related to familiar screening are not presented in this manuscript, since it is not fully completed yet.

### 2.2. Study Population

Cases of SD between 1 and 50 years old, with findings of uncertain significance in the autopsy, were included. These include idiopathic LVH, defined as unexplained LVH (heart weight >500 g in males and >400 g in females) in the absence of myocardial disarray or secondary causes of LVH [13]; and/or CA without significant narrowing of the arterial lumen (obstruction inferior to 75% of the vessel) with no evidence of acute or chronic ischaemia11; and/or PMF without signs of structural or ischemic cardiopathy [11,14]. PMF could be focal or spread and patchy.

Cases of SD aged ≤1 year old or above 50 years old were excluded, as well as those with a definitive cause of death identified. Cases with no clear cause of death in the post-mortem evaluation, known as sudden unexplained death syndrome (SUDS), were also excluded [9,10]. Cases in which toxicological studies were positive for illicit drugs, alcohol, or medical drugs in doses above the therapeutic range were excluded. Finally, when coronary disease affected ≥75% of the lumen, or showed evidence of acute or chronic ischemia, cases presenting ventricular hypertrophy associated to a secondary cause as severe arterial hypertension, or evidence of myocardial disarray, and cases presenting myocardial fibrosis related to structural or ischemic cardiac diseases, were also excluded [17].

### 2.3. Autopsy

All decedents underwent a full autopsy evaluation performed by a local pathologist at the Balearic Institute of Legal Medicine according to forensic guidelines [8]. Following the exclusion of extra-cardiac causes, the heart was referred to the Histopathology Department at the National Institute of Toxicology and Forensic Science (Barcelona, Spain). Macroscopic examination of the heart and histological analysis was performed, all cardiac structures were systematically examined, and a toxicology screening was conducted in all cases. A blood sample from the decedent was sent to the genetic lab for the molecular autopsy, following the autopsy results.

### 2.4. Clinical Information in Decedents

The referring forensic pathologist was requested to provide information about the demographics of the deceased, past medical and family history, cardiac symptoms, the nature and level of physical activity, and the exact circumstances of death. Information collected was completed by seeking data available from the local computerized medical history. Exceptions included foreign cases who did not have a local medical history.

### 2.5. Statistical Analysis

A descriptive study was performed by calculating the frequencies of the qualitative variables, the mean ± standard deviation, and the median and interquartile range for the quantitative variables. Data analysis was undertaken using the SPSS^®^ (Chicago, IL, USA) 15.0 software.

### 2.6. Institutional Review Board Statement

The study was conducted according to the guidelines of the Declaration of Helsinki and approved by the Ethics Committee of the “Autonomous Committee of Ethical in Clinical Investigation of the Balearic Islands”: project identification code IB 1525/11 PI, date of approval 23 February 2011.

## 3. Results

A total of 195 cases of SD, between 1 and 50 years old, were registered at the MUSIB program from February 2015 to January 2020. We analyzed cases that presented a complete forensic pathologist report with histological, toxicological and genetic testing results. Among the 195 cases of SD, we excluded 42 incomplete cases and 65 cases with a definitive cause of death. Among the remaining cases, 57 were excluded following the exclusion criteria: 30 cases (34%) showed positive results for the toxicological screening; one case (1%) presented records of severe arterial hypertension as a secondary cause for LVH; 11 cases (13%) presented CA affecting more than 75% of the vessel lumen, and five cases (6%) were younger than 1 year old. Finally, 10 cases (11%) showing no pathological findings in the autopsy were also excluded and were considered as the SUDS group.

**Figure 1:**
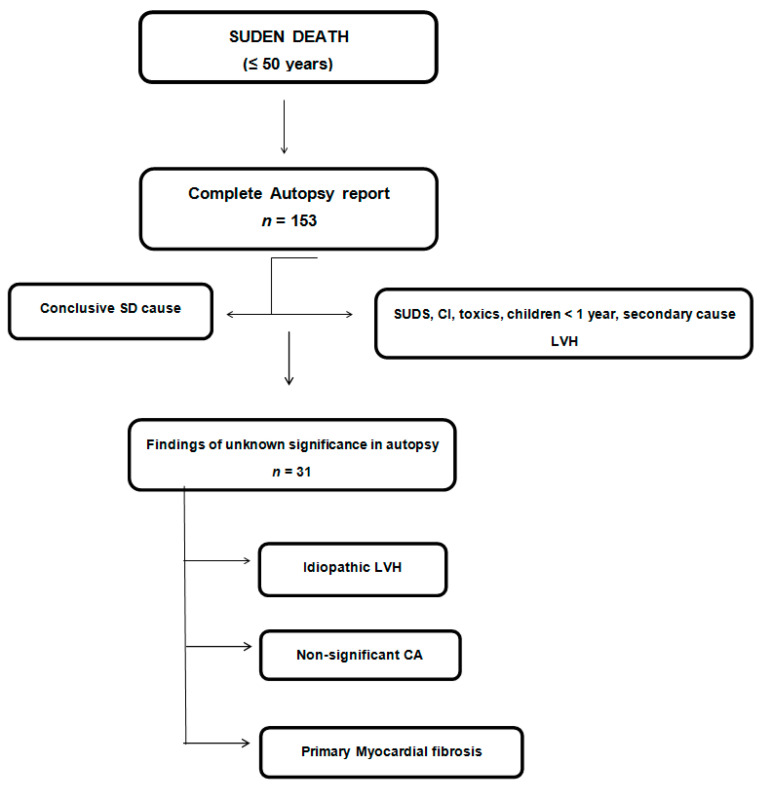
Flow chart of study cohort. SD, sudden death. SUDS, sudden unexpected death syndrome. IC, ischemic cardyomyopathy. LVH, left ventricular hypertrophy. CA, coronary atherosclerosis.

A total of 31 cases fulfilling the pre-established inclusion criteria were further investigated in the present study (Figure 1).

Note that patients with an autopsy result showing an inherited heart disease or SUDS were also genetically tested, but it is not shown in this article.

### 3.1. Demographic and Clinical Characteristics of Decedents

The mean age of death was 41.06 ± 7.21 years (range, 17–50 years). Most cases were male (*n* = 25, 80.65%), and 64.52% were resident in the Balearic Islands (*n* = 20). Related to the cardiovascular risk factors identified, smoking was the most frequent one representing 32.26% (*n* = 10) of the cases, followed by obesity in 22.58% (*n* = 7), and arterial hypertension and dyslipidemia was present in 19.35% (*n* = 6) each. Most cases did not practice any sport (64.52%, *n* = 20) and the most frequent circumstance of death was at rest (*n* = 15, 48.39%).

History of myocardium disease or SD in the family was proven in one case (3.23%), considering that the precedent was unknown in 12 cases (39%), who were foreigners.

The most frequent symptom during the onset of the SD event was syncope in a 19.35% of the cases (*n* = 6). The circumstances of SD were unknown in 13 cases (41.94%). Demographic and clinical characteristics are presented in Table 1.

### 3.2. Autopsy Findings

**Figure 2:**
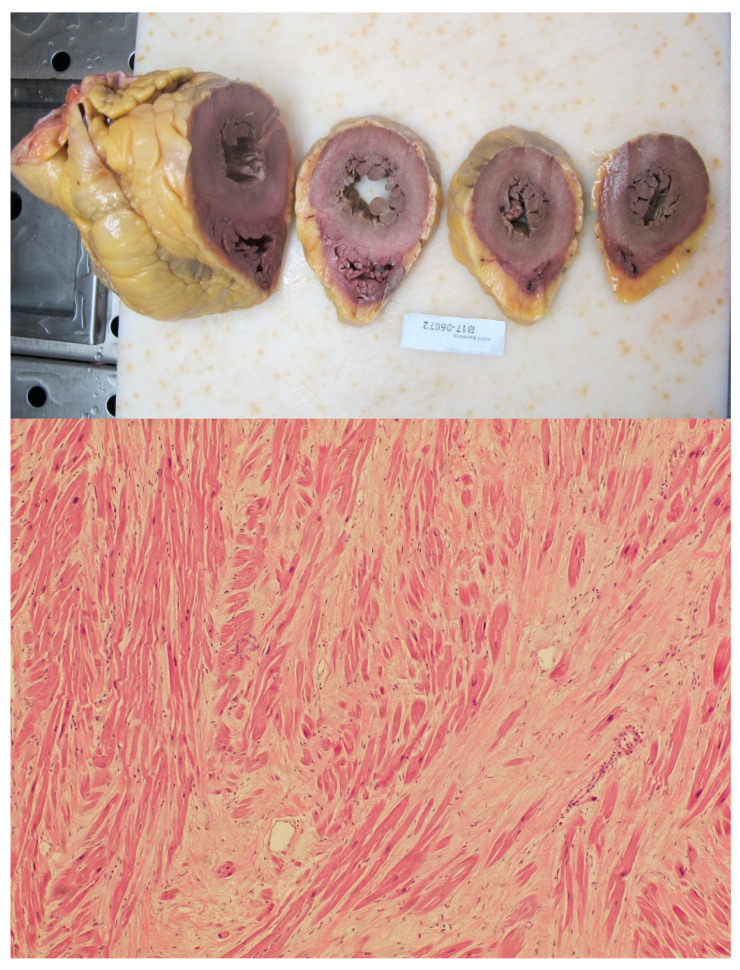
Macroscopic examination of the heart of a sudden death decedent showing left ventricular hypertrophy (**above**); histological analysis of the heart of another sudden death decedent at 60× showing the presence of myocardial fibrosis in the absence of disarray (**below**).

The post-mortem findings in the 31 cases included, showed that 51.61% (*n* = 16) presented idiopathic LVH, 54.84% (*n* = 17) had CA without significant narrowing of the arterial lumen, and 77.42% (*n* = 24) had PMF: one case (3.23%) presented isolated LVH, four (12.9%) showed isolated CA and five cases (16.13%) had isolated MF. The three findings were present in 16.13% (*n* = 5), 6.46% (*n* = 2) showed CA and LVH without PMF, and 25.8% (*n* = 8) showed LVH together with PMF without CA (Figure 2).

When analyzing the CA group (*n* = 17), we found that 82.35% (*n* = 14) had only one vessel affected. Among these, 85.71% (*n* = 12) showed involvement of the left anterior descending artery, one case (7.14%) had right coronary involvement, and one case (7.14%) affected the circumflex artery. There was affection of three vessels in 17.64% (*n* = 3) of the cases (Table 2).

### 3.3. Molecular Autopsy

**Figure 3:**
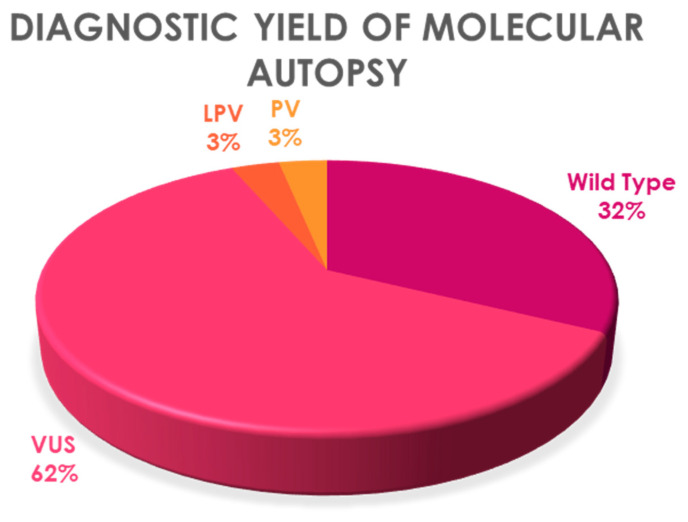
Molecular autopsy results. VUS, variant of unknown significance; PV, pathogenic variant; LPV, likely pathogenic variant.

Genetic testing was performed in the 31 cases included. It was negative and labeled as wild type in 32.25% (*n* = 10). The remaining 67.74% (*n* = 21) were positive for some of the variants of interest: VUS 61.29% (*n* = 19), LPV 3.23% (*n* = 1), and PV 3.23% (*n* = 1). The yield of molecular autopsy decreased to 6.45% when taking into account the PV and LPV (*n* = 2). Genes involved were structural genes in 41.93% (*n* = 13), and related to channelopathies in 38.7% (*n* = 12) (Figure 3).

The first case showing positive genetic testing was a 46 year old man from Portugal, who presented SD preceded by abdominal pain. The molecular autopsy identified a heterozygosis mutation in the MYBPC3 gene: c.2176C > T (p.Arg726Cys). This variant was previously described as PV associated with hypertrophic cardiomyopathy25 in a 68 year old woman, and to SD in cases of dilated cardiomyopathy26, within a range of penetrance between 41 and 50 years old cases. It is classified as LPV (class 4) according to ACMG recommendations. Additionally, two VUS in the genes TPM1 (related to hypertrophic cardiomyopathy) and LDB3 (related to hypertrophic cardiomyopathy and dilated cardiomyopathy) were detected in the genetic testing. The autopsy only revealed nonsignificant CA in the left anterior descending artery as a finding of unknown significance, thus no relationship between molecular and tissue autopsy findings could be established as a cause of death.

The second case with a positive result in the genetic testing was a 50 year old man who presented the event of SD while sleeping. The autopsy revealed an oversized heart with a weight of 470g without signs of disarray; mild PMF, and CA of less of 25% of the artery lumen in each epicardial artery. The molecular autopsy showed a heterozygous PV in the MYBPC3 gene: c.2670dup (p.Arg891Alafs*160), which produces a change in the reading frame that starts at codon 891 and ends in a stop codon 159 codons later. This variant is classified as PV according to the ACMG recommendations.

All remaining genetic results and the associations between genetic variants observed and diseases previously reported in literature are presented in Table 3.

## 4. Discussion

Post-mortem histopathological evaluation may identify abnormalities in cardiac structures, which may not be sufficient to reach a precise diagnosis in SD events [18]. These findings of uncertain significance include idiopathic LVH, noncritical CA, and PMF, among others. Their relationship with SD has not yet been proven, they may represent an incidental finding of no relevance, or may be a risk factor for SD. Their true etiological impact must still be elucidated.

The appearance of NGS in genetic testing has allowed the study of a broad number of genes and improvements in the clinical investigation related to genetic cardiopathy was possible, allowing to reach a clear etiological diagnosis in some cases of SD.

Therefore, we contemplate whether it is useful to perform molecular autopsy in cases of SD with autopsy findings of uncertain significance, considering that genetic testing is never 100% effective, even in cases of well-established myocardium disease [19].

We present a prospective descriptive study of molecular autopsy in cases of SD between 1 and 50 years with autopsy findings of unknown significance, to evaluate the diagnostic yield of genetic testing.

Concerning idiopathic LVH, its pathogenicity and origin is unknown. Several hypotheses have suggested that it is a continuous spectrum in hypertrophic myocardium disease, which would increase mortality risk independently from other factors. The increase in myocardium mass could reduce myocardial perfusion and thus increase susceptibility to ischemia and risk of ventricular arrhythmia [11,14,17]. Other hypotheses suggest that there may be local and patchy focuses of disarray that may not be detected by traditional autopsy methods [11].

In relation to CA with a nonsignificant lumen narrowing, some hypotheses suggest that even mild obstruction might be enough to generate ischemia and unmask underlying electric disorders related to hereditary arrhythmogenic disorders [11]. Additionally, spontaneous autolysis of the thrombus might occur and the absence of ischemic signs may be related to insufficient time for them to develop.

As regards PMF, it has been associated with ventricular arrhythmia due to a re-entrant mechanism by several groups [11,14]. Some hypotheses relate PMF to cell degeneration due to hypoxia, or spontaneous degeneration due to an underlying cause as an alternative phenotypic expression pathway in specific inherited structural diseases, like hypertrophic myocardial disease [17,20]. PMF has also been reported in elite athletes [11]. Even though findings of unknown significance could be an illness precursor, they may also simply be a cardiac phenomenon related to cell aging.

In the series we analyzed, the diagnostic yield rate of genetic testing was 67.74% including VUS, LPV and PV. The rate decreases to 6.45% if only considering PV and LPV, which indeed is a low diagnostic yield. The two positive genetic test results obtained in our series corresponded to different mutations in the MYBPC3 gene as previously described, which have been mainly related to hypertrophic myocardium disease in the literature. When analyzing autopsy results, we find that one of the cases with the c.2176C > T (p.Arg726Cys) genetic variant in the MYBPC3 gene, was a 46 year old man, with no signs of hypertrophic myocardium disease, neither of idiopathic LVH, and who only presented nonsignificant CA in one vessel. The same genetic mutation has been previously described in a 68-year-old woman in the literature [21], what may suggest that the disease may have not have complete penetrance by the age of our case. The second case we mentioned with a positive result involving the c.2670dup (p.Arg891Alafs*160) mutation was a 50 year-old man, whose autopsy revealed the presence of idiopathic LVH, nonsignificant CA and PMF, but the pieces analyzed had no sign of myocardium disarray, and no secondary cause of myocardium hypertrophy or PMF was detected. The same genetic variant was previously described in the literature affecting a 44 year old man [21], therefore, we may assume that by the age of our case the hypertrophic myocardium disease would have been already developed. One thing to mention is the enormous advantage that would have been obtained by carrying on familiar screening in these two cases, since really useful information could result from making a close clinical and genetic evaluation of first line relatives.

Similar series previously published by Papadakis et al. [14] evaluated 157 blood relatives between 4 and 64 years of 41 SD cases with cardiac findings of unknown significance in the cardiac autopsy, even though genetic test in the SD cases was not available. They diagnosed a probable or definitive hereditary underlying cause of SD in 21 of the 41 cases (51%). They made a comparison between the diagnostic yield of this subgroup (51%) and the subgroup of 163 cases of SUDS (47.2%). Both showed an underlying cause of primary arrhythmogenic syndrome as the main diagnosis. They also analyzed the yield of genetic testing in the relatives: 24.6% for the SUDS and 22.9% in the subgroup with findings of uncertain significance. These results show a considerable higher diagnostic yield of genetic testing when compared to ours and more cases of genetic variants related to channelopathies were identified by them, what may be explained by the larger series of cases they included in a completely different study design.

A similar study was published by Lahrouchi et al. [22], who evaluated the diagnostic yield of molecular autopsy in cases of SD with cardiac findings of unknown significance and cases of SD with a clear diagnosis of cardiopathy. They obtained similar rates between the subgroup of decedents diagnosed of cardiopathy and the group of alive patients with cardiopathy: close to 32%. However, genetic testing in the subgroup of SD with cardiac findings of uncertain significance had a low diagnostic yield of 3% close to the results we obtained in our series, taking into consideration that they presented a small series of 28 cases similar to us.

Another reference is to be made regards the study published by Juntilla et al. [23] in which they evaluated the association between SD cases with only PMF in the autopsy and genetic disorders predisposing to myocardial fibrosis or ion channelopathies. They analyzed 145 cases and reported the detection of potentially relevant genetic variants in 26 subjects (27%), 10% were classified as PV or LPV, and the predominant variants detected were related to myocardial structure-coding genes, mainly arrhythmogenic right ventricular cardiomyopathy, dilated cardiomyopathy and hypertrophic cardiomyopathy.

Finally, Hertz et al. [15] studied 61 cases of SUDS and informed that 34% of the cases (*n* = 21) had 25 genetic variants that were classified as having likely functional effects: 40% of these were related to genes previously associated to cardiomyopathies, and 60% were associated to cardiac channelopathies. When analyzing the SUDS subgroup in our cohort (*n* = 10), genetic testing was positive in two cases: one for a PV and the other for an LPV. This estimates a genetic yield of 20% for our small series of SUDS. The different results they obtained may be related to the larger series of SUDS cases analyzed by them (61 vs. 10).

NGS provides a rapid, cost-effective, and massive gene analysis [9]. However, the interpretation of the results is challenging, since VUS appear to be the main finding [19,24,25], and in many cases medical history may be unavailable as well as autopsy results may be inconclusive, as reflected in our series.

The molecular autopsy should be a tool to complement clinical evaluation, and never replace it [26]. The genetic yield is never 100%, it supports the diagnosis when there is a clear phenotype, while a negative test does not exclude disease.

We believe it is necessary to be cautious when interpreting molecular autopsy results and always relate them to the clinical and familial context.

### Limitations

The main limitations of the present manuscript consist of a small number of cases, including a sample with great proportion of foreigners, which determines an important loss of data related to the medical history of cases and the subsequent lack of information as regards family screening.

## 5. Conclusions

Molecular autopsy in SD cases between 1 and 50 years old, with cardiac findings of uncertain significance, has a low diagnostic yield in our small series of cases, being VUS the most frequent variant observed. Larger studies focusing on the evaluation of the utility of molecular autopsy in this population are needed to reach more robust results and to help to elucidate this issue. This is relevant for diagnostic purposes, and as guidance for familial screening to detect healthy carriers and affected ones. Clinical relevance of the VUS and their relation to disease is another relevant aspect that needs to be elucidated considering their frequent appearance in genetic test results.

To conclude, we consider that molecular autopsy could be profitable in the context of clear conditions for its indication.

## Figures and Tables

**Table 1:** Demographic and clinical characteristics of decedents. SD, sudden death.

Demographic and Clinical Characteristics	
**Total *n***	31
Men	80.65% (*n* = 25)
Age	41.06 ± 7.21 (17–50 years old)
Smoking	32.26% (*n* = 10)
Former smoking	6.45% (*n* = 2)
Obesity	22.58% (*n* = 7)
Arterial hypertension	19.35% (*n* = 6)
Dyslipidemia	19.35% (*n* = 6)
Diabetes	3.23% (*n* = 1)
Family history of SD	3.23% (*n* = 1)
Atrial fibrillation	--
Sports practice	12,9% (*n* = 4)
History of psychiatric disorder under treatment	32.26% (*n* = 10)
History of psychiatric disorder without treatment	3.23% (*n* = 1)
Local resident	64.52% (*n* = 20)
**Circumstance of Death**	
Rest	48.39% (*n* = 15)
Exertion	22.58% (*n* = 7)
Unknown	19% (*n* = 6)
Sleep	6.45% (*n* = 2)
Post exertion	3.23% (*n* = 1)
**Symptoms**	
Syncope	19.35% (*n* = 6)
Angina	12.9% (*n* = 4)
Abdominal pain	9.68% (*n* = 3)
Asymptomatic	6.45% (*n* = 2)
Dyspnea	3.23% (*n* = 1)
Dizziness	3.23% (*n* = 1)
Palpitations	--
Seizures	--
Unknown	41.94% (*n* = 13)

**Table 2:** Description of autopsy findings of uncertain significance. LVH, left ventricular hypertrophy; CA, coronary arteriosclerosis; PMF, primary myocardial fibrosis; LCA, left coronary artery; LAD, left anterior descending artery; Cx, circumflex artery; RCA, right coronary artery.

Autopsy Findings of Uncertain Significance	
**Idiopathic LVH**	51.61% (*n* = 16)
Nonsignificant CA	54.84% (*n* = 17)
PMF	77.42% (*n* = 24)
Isolated idiopathic LVH	3.23% (*n* = 1)
Isolated PMF	16.13% (*n* = 5)
Isolated CA	12.9% (*n* = 4)
Idiopathic LVH, CA y PMF	16.13% (*n* = 5)
Idiopathic LVH and CA	6.46% (*n* = 2)
CA and PMF	19% (*n* = 6)
Idiopathic LVH and PMF	25.8% (*n* = 8)
**Nonsignificant Coronary Atherosclerosis**	
Number of vessels affected	
1 2 3	82.35% (*n* = 14) -- 17.64% (*n* = 3)
Coronary artery affected	
LCA LAD Cx RCA Three vessel affection	-- 85,71% (*n* = 12) 7,14% (*n* = 1) 7,14% (*n* = 1) 17,64% (*n* = 3)
Lumen narrowing	
<50% 50–75% >75%	47% (*n* = 8) 53% (*n* = 9) --

**Table 3:** Characteristics of Victims of Sudden Cardiac Death with Autopsy Findings of Uncertain Significance. M, male. F, female. LVH, left ventricular hypertrophy. CA, coronary atherosclerosis. MF, myocardial fibrosis. WT, wild type. HCM, hypertrophic cardiomyopathy. DCM, dilated cardiomyopathy. LQTS, long QT syndrome. ACM, arrythmogenic cardiomyopathy.

Case	Age	Sex	SD Cause	Autopsy Finding of Uncertain Significance	Affected Gene	Mutation	Mutation Classification ACMG	Mutation Related Disease	Sports Practice	SD Circumstances	SD or Cardiopathy Family History
4	37	M	Inconclusive	LVH, MF	WT	--	--	--	No	Rest	*n*/d
11	32	M	Inconclusive	MF	SCN5A	Gln692Lys	VUS	LQTS	No	Rest	No
					SLMAP	IVS8-4G>A	VUS	Brugada Syndrome			
17	44	M	Inconclusive	LVH, CA, MF	TTN	p.Pro12648Leu	VUS	DCM o HCM	Yes	Activity	No
18	43	F	Inconclusive	CA	SPRED1	Ser40Thr	VUS	Legius Syndrome	No	Rest	*n*/d
23	41	M	Inconclusive	LVH, CA	JUP, NOTCH1, NPPA, PLEC y TTN.	JUP (p.E453D); NOTCH1 (p.T123M); NPPA (p.*152Argext*?); PLEC(p.R1578C); TTN (p.Y28626C)	VUS	Arrhythmogenic cardiomyopathy (JUP); Vao disease (NOTCH1); Atrial fibrillation and HCM(NPPA); muscular dystrophy (PLEC); DCM o HCM (TTN)	No	Rest	No
34	47	F	Idiopathic LVH	LVH, MF	WT	--	--	--	No	Rest	No
38	38	F	Inconclusive	LVH, CA	SCN5A	p.E428K	VUS	Brugada Syndrome, Familiar CDM	*n*/d	Activity	*n*/d
					SMAD9	p.L22L	VUS	Hereditary pulmonar hypertension type 2			
					ACVRL1	c.-4G>T	VUS	Hereditary hemorrhagic telangiectasia type 2			
					ALMS1	p.S1636N	VUS	Alstrom syndrome			
					TTN	p.R15268C	VUS	DCM, HCM, Muscular Dystrophy			
					TNXB	p.G4242V	VUS	Ehlers-Danlos type III			
					OBSL1	p.R419C	VUS	3M syndrome			
40	47	M	Idiopathic LVH	LVH, CA, MF	ANK3, PLEC, SGCD, TNXB y TTN.	ANK3 (p.R4369Q); PLEC (p.A1554V); SGCD (p.R31Q); TNXB (p.G4048S); TTN (p.G25650S)	VUS	Brugada syndrome (ANK3); muscular dystrophy (PLEC y SGCD); DCM (SGCD); Ehlers-Danlos (TNXB); DCM o HCM (TTN)	*n*/d	Rest	*n*/d
46	42	M	Inconclusive	CA	MYH6	p.Met1237Leu	VUS	HCM, atrial septal deffect	*n*/d	n/d	*n*/d
					DMD	p.Lys1004Glu	VUS	Duchenne and Becker muscular dystrophy			
62	38	M	Idiopathic LVH	LVH, MF	FLNC	p.Pro632	VUS	HCM, DCM, RCM, muscular dystrophy	*n*/d	Rest	Yes
					KCNE1	p.Val47lle	VUS	LQTS			
64	43	M	Idiopathic LVH	LVH, MF	NEBL	p.Tyr57His	VUS	CDM, Endomiocardial fibroelastosis	No	n/d	No
69	40	M	Idiopathic LVH	LVH, CA, MF	WT	--		--	*n*/d	Activity	No
73	49	F	Inconclusive	MF	WT	--	--	--	No	n/d	No
74	36	M	Idiopathic LVH	LVH, MF	JUP	p.Val159Leu	VUS	Arrhythmogenic cardiomyopathy	No	Rest	No
					PKP2	p.Arg490Trp	VUS	Arrhythmogenic cardiomyopathy			
82	38	F	Idiopathic LVH	LVH, MF	WT	--	--	--	No	Activity	*n*/d
93	29	M	Idiopathic LVH	LVH, MF	gen DSG2	p.Thr335Ala	VUS	Arrhythmogenic cardiomyopathy	*n*/d	n/d	*n*/d
98	46	M	Inconclusive	EC	TPM1	p.Arg21Leu	VUS	HCM	No	Rest	*n*/d
					LDB3	p.Phe501Ser	VUS	DCM, HCM			
					MYBPC3	p.Arg726Cys	LPV	HCM			
99	17	M	Inconclusive	MF	TTN	pThr34494Pro	VUS	DCM, Arrhythmogenic cardiomyopathy, HCM, myopathy, muscular dystrophy	No	Activity	No
101	40	M	Inconclusive	LVH, CA	WT	--	--	--	No	Rest	No
102	49	M	Inconclusive	LVH, CA, MF	WT	--	--	--	No	Rest	No
105	50	M	Idiopathic LVH	LVH, CA, MF	MYBPC3.	p.Arg891Alafs*160	PV	HCM	No	Sleep	No
109	45	M	Inconclusive	LVH, MF	WT	--	--	--	No	Rest	*n*/d
119	47	M	Inconclusive	CA, MF	RyR2	c.13328+3A>G	VUS	Catecholaminergic cardiomyopathy, arrhythmogenic cardiomyopathy	No	n/d	No
					RyR2	p.Arg4573His	VUS	Catecholaminergic cardiomyopathy, arrhythmogenic cardiomyopathy			
					RBM20	p.His412Asp	VUS	DCM			
145	35	M	Idiopathic LVH	LVH	WT	--	--	--	Yes	Activity	No
154	49	M	Inconclusive	CA, MF	MYH6	p.Ala1004Ser	VUS	DCM, HCM	n/d	n/d	*n*/d
157	35	M	Inconclusive	CA, MF	TTN	p.Arg33104Cys	VUS	DCM, arrhythmogenic cardiomyopathy, HCM, myopathy, muscular dystrophy	No	Sleep	No
163	52	M	Inconclusive	CA, MF	ANK2	p.Thr2395Pro	VUS	LQTS	Yes	After activity	No
166	39	F	Inconclusive	MF	WT	--	--	--	No	*n*/d	*n*/d
172	49	M	Inconclusive	CA, MF	TRPM4	p.Ala1076Thr	VUS	Brugada Syndrome, Cardiac heart block	No	Rest	*n*/d
